# Supplementary material for: N-Terminal Acetyltransferases Are Cancer-Essential Genes Prevalently Upregulated in Tumours
Source: Cancers (Basel). 2020 Sep 15;12(9):2631. doi: 10.3390/cancers12092631 (PMC7565035; doi:10.3390/cancers12092631)
Supplement: Supplementary file 1 [file cancers-12-02631-s001.pdf]

Supplementary Figure Legends

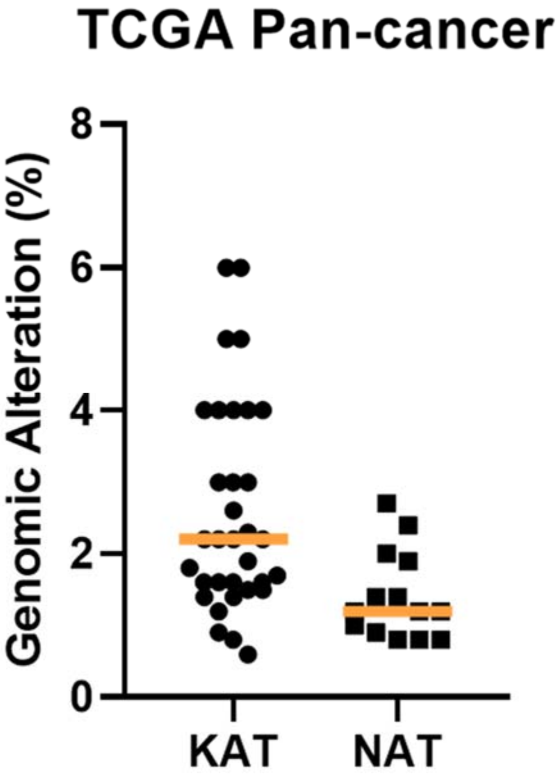

Figure S1. Comparison of genomic alteration frequency-both copy number alterations and mutations-for lysine acetyltransferases (KATs) and N-terminal Acetyltransferases (NATs) across TCGA pan-cancer study of 32 tumour types. Each circle represents one gene.

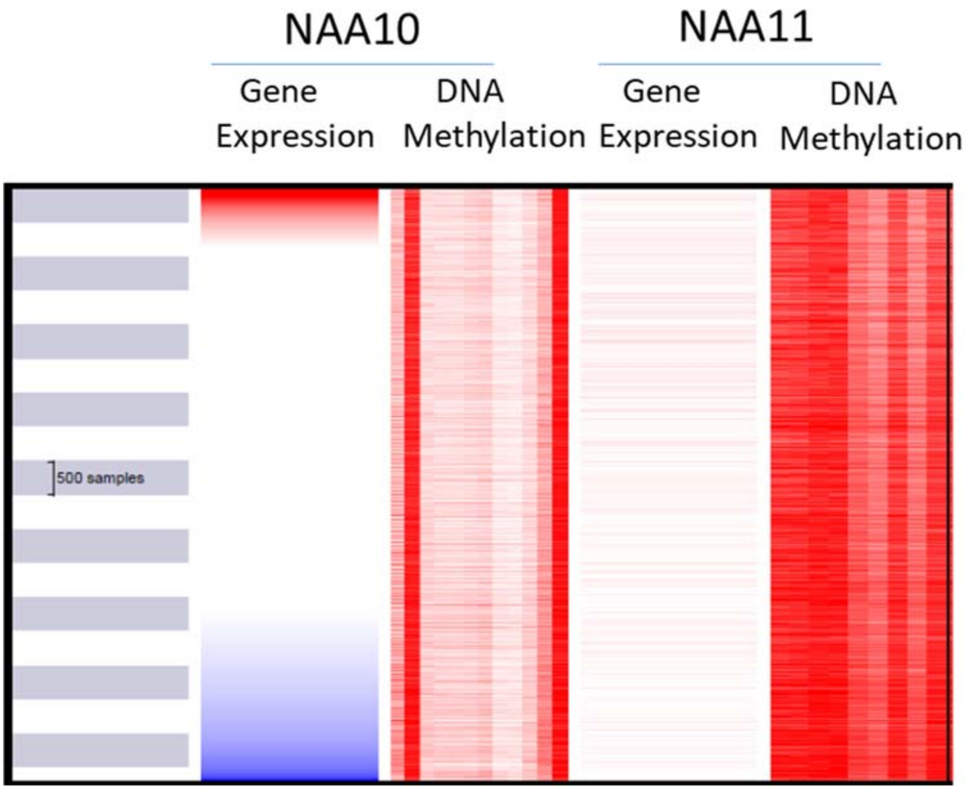

Figure S2. A multi-omic comparison of DNA methylation and expression of NAA11 and NAA10 across TCGA pan-cancer study of 32 cancer types. Darker red colour indicates higher values of gene expression or methylation of DNA probes.

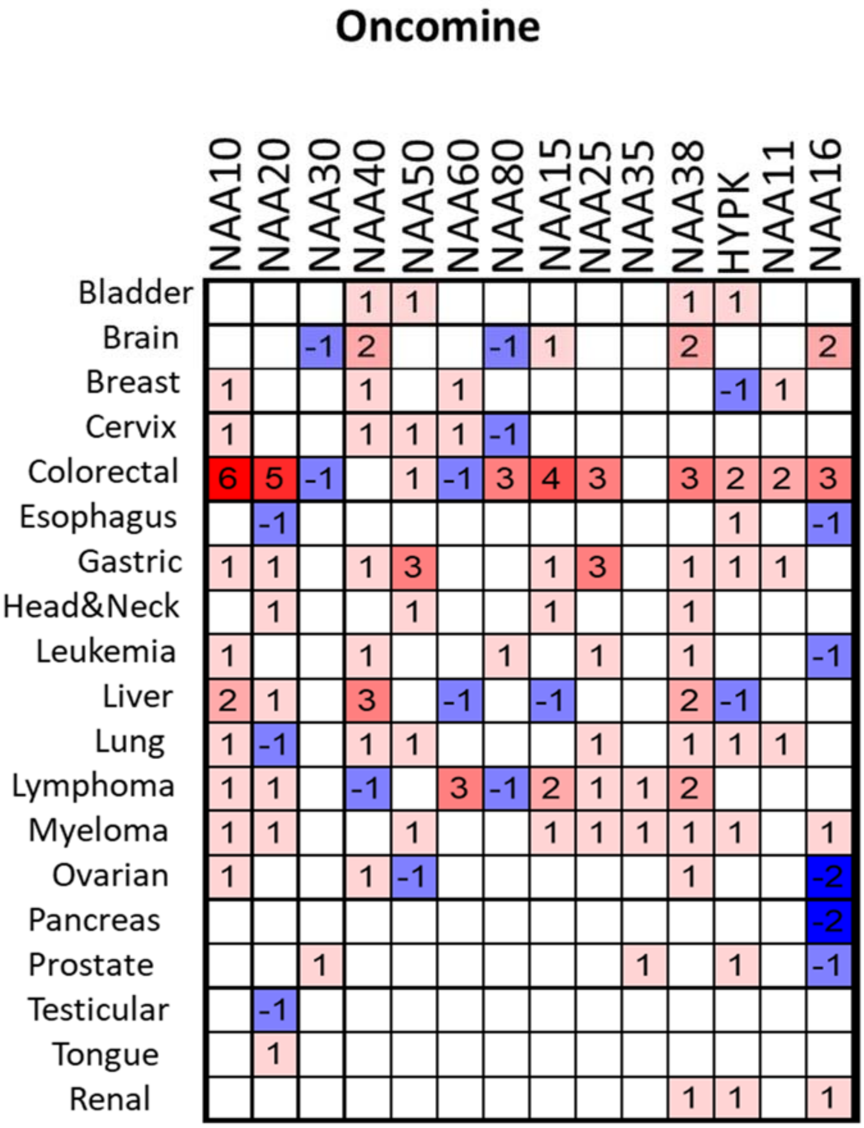

Figure S3. Examination of altered NAT transcript in the Oncomine microarray dataset for tumours with altered expression compared to normal tissues. Positive numbers within boxes indicate the number of studies with significant upregulation of NATs. Negative number within boxes indicate studies with downregulation of NATs.

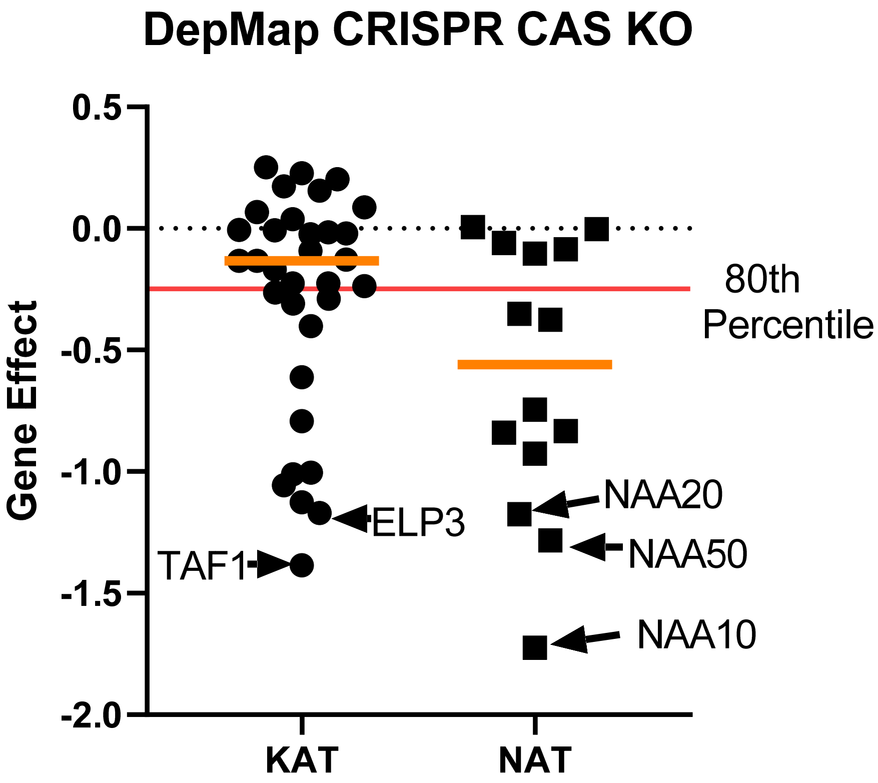

Figure S4. Comparison of dependency of cancer cells on KAT and NAT genes. Plotter are average gene effect (CEREC) of CRISPR-CAS KO across 739 cell lines of DepMap project. More negative values indicate greater sensitivity to KO. Each gene is represented by a symbol and the median gene effect across all cell lines is depicted by an orange line.

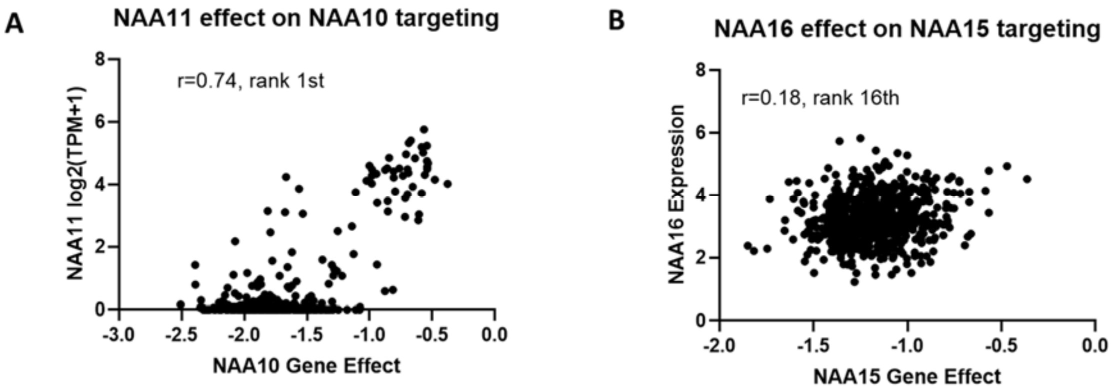

Figure S5. NAA11 and NAA16 transcript levels are associated with reduced sensitivity to targeting of their paralogues NAA10 and NAA15 in the DepMap CRISPR CAS KO project. Plots depict scatterplot of expression of NAA11 and NAA16 vs sensitivity of cell lines to targeting of their paralogues. Pearson's correlation was calculated and rank is the relative rank of the correlation of sensitivity to KO of that NAT across all measured transcripts.

Supplementary Tables

Table S1. Histone H2A missense mutations of "SRGR" motif in TCGA pan-cancer.

Histone	Sample	Tumour	Mutation
HIST1H2AG	TCGA-DD-AADV-01	Hepatocellular Carcinoma	S1C
HIST1H2AI	TCGA-77-A5GH-01	Lung Squamous Cell Carcinoma	S1C
HIST1H2AM	TCGA-CV-A6JU-01	Head and Neck Squamous	S1C
HIST1H2AM	TCGA-FG-A710-01	Oligodendroglioma	S1C
HIST2H2AC	TCGA-EP-A3JL-01	Hepatocellular Carcinoma	S1C
HIST3H2A	TCGA-ZF-A9R9-01	Bladder Urothelial Carcinoma	S1C
HIST1H2AI	TCGA-QK-A8Z7-01	Head and Neck Squamous	S1F
HIST1H2AK	TCGA-A2-A0CT-01	Breast Invasive Ductal Carcinoma	S1L
HIST1H2AK	TCGA-AG-A01J-01	Rectal Adenocarcinoma	S1L
HIST2H2AB	TCGA-BB-4223-01	Head and Neck Squamous	S1L
HIST1H2AC	TCGA-OL-A66J-01	Breast Invasive Lobular Carcinoma	S1P
HIST2H2AB	TCGA-VQ-A91K-01	Intestinal Type Stomach Adenocarcinoma	S1P
HIST1H2AL	TCGA-D8-A1XQ-01	Breast Invasive Ductal Carcinoma	S1T
HIST1H2AI	TCGA-JY-A6FA-01	Esophageal Squamous Cell Carcinoma	S1Y
HIST1H2AJ	TCGA-AX-A2HC-01	Uterine Endometrioid Carcinoma	S1Y
HIST1H2AD	TCGA-D7-A4YV-01	Tubular Stomach Adenocarcinoma	G2E
HIST1H2AE	TCGA-RS-A6TP-01	Head and Neck Squamous Cell Carcinoma	G2E
HIST1H2AI	TCGA-RD-A8N9-01	Diffuse Type Stomach Adenocarcarcinoma	G2E
HIST1H2AA	TCGA-FW-A3TU-06	Cutaneous Melanoma	G2R
HIST1H2AG	TCGA-QQ-A5VC-01	Leiomyosarcoma	G2R
HIST1H2AG	TCGA-24-1417-01	Serous Ovarian Cancer	G2V
HIST1H2AH	TCGA-B6-A0RS-01	Breast Invasive Ductal Carcinoma	R3C

Histone	Sample	Tumour	Mutation
HIST1H2AL	TCGA-AA-3947-01	Mucinous Adenocarcinoma of the Colon	R3C
HIST3H2A	TCGA-HU-A4GF-01	Tubular Stomach Adenocarcinoma	R3C
HIST2H2AC	TCGA-E7-A5KF-01	Bladder Urothelial Carcinoma	R3C
H2AFY	TCGA-HU-A4GX-01	Diffuse Type Stomach Adenocarcinoma	R3C
HIST2H2AC	TCGA-E7-A5KF-01	Bladder Urothelial Carcinoma	R3C
HIST1H2AC	TCGA-33-AASI-01	Lung Squamous Cell Carcinoma	R3H
HIST1H2AI	TCGA-CM-4743-01	Colon Adenocarcinoma	R3H
HIST2H2AC	TCGA-N5-A4RD-01	Uterine Carcinosarcoma..	R3H
HIST1H2AA	TCGA-63-A5MI-01	Lung Squamous Cell Carcinoma	R3L
HIST1H2AM	TCGA-60-2723-01	Lung Squamous Cell Carcinoma	R3L
HIST1H2AA	TCGA-EE-A29M-06	Cutaneous Melanoma	R3Q
HIST1H2AA	TCGA-EE-A3J8-06	Cutaneous Melanoma	R3Q
HIST1H2AA	TCGA-FS-A1ZK-06	Cutaneous Melanoma	R3Q
HIST1H2AA	TCGA-WE-AAA4-06	Cutaneous Melanoma	R3Q
HIST1H2AG	TCGA-DK-AA6R-01	Bladder Urothelial Carcinoma	G4C
HIST2H2AC	TCGA-N5-A4RD-01	Uterine Carcinosarcoma	G4C
HIST1H2AM	TCGA-66-2759-01	Lung Squamous Cell Carcinoma	G4S
HIST1H2AG	TCGA-DK-AA6R-01	Bladder Urothelial Carcinoma	G4V
HIST1H2AH	TCGA-V5-A7RC-01	Esophageal Squamous Cell Carcinoma	G4V
HIST1H2AJ	TCGA-CD-8531-01	Diffuse Type Stomach Adenocarcinoma.	G4V

Table S2. Histone H4 missense mutations of "SRGR" motif in TCGA pan-cancer.

Histone	Sample	Tumour	Mutation
HIST1H4H	TCGA-DK-A3WW-01	Bladder Urothelial Carcinoma	S1C
HIST1H4K	TCGA-CH-5794-01	Prostate Adenocarcinoma	S1C
HIST1H4A	TCGA-24-2280-01	Serous Ovarian Cancer	S1F
HIST1H4B	TCGA-IR-A3LK-01	Cervical Squamous Cell Carcinoma	S1F
HIST1H4E	TCGA-OR-A5LF-01	Adrenocortical Carcinoma	S1F
HIST4H4	TCGA-19-5956-01	Glioblastoma Multiforme	S1Y
HIST1H4J	TCGA-DD-A4NV-01	Hepatocellular Carcinoma	G2S
HIST1H4D	TCGA-GC-A3RC-01	Bladder Urothelial Carcinoma	G2A
HIST1H4D	TCGA-CC-A7IE-01	Hepatocellular Carcinoma	G2A
HIST1H4F	TCGA-73-A9RS-01	Lung Adenocarcinoma	G2V
HIST1H4J	TCGA-CQ-5332-01	Head and Neck Squamous Cell Carcinoma	G2V
HIST1H4I	TCGA-EO-A22U-01	Uterine Endometrioid Carcinoma	R3C
HIST1H4J	TCGA-J9-A8CM-01	Prostate Adenocarcinoma	R3C
HIST1H4J	TCGA-DX-A8BU-01	Undifferentiated Pleomorphic Sarcoma	R3C
HIST1H4K	TCGA-VQ-A94R-01	Intestinal Type Stomach Adenocarcinoma	R3C
HIST1H4K	TCGA-ZB-A96V-01	Thymoma	R3C
HIST1H4B	TCGA-B3-8121-01	Papillary Renal Cell Carcinoma	R3G
HIST1H4J	TCGA-DJ-A1QG-01	Papillary Thyroid Cancer	R3G
HIST1H4F	TCGA-LD-A74U-01	Breast Invasive Carcinoma (NOS)	R3K
HIST4H4	TCGA-GU-AATO-01	Bladder Urothelial Carcinoma	R3Q
HIST1H4E	TCGA-91-6829-01	Lung Adenocarcinoma	G4C
HIST1H4B	TCGA-Z2-AA3S-06	Cutaneous Melanoma	G4D
HIST1H4J	TCGA-BR-6709-01	Stomach Adenocarcinoma	G4D
HIST1H4E	TCGA-CR-5247-01	Head and Neck Squamous Cell Carcinoma	G4S
HIST1H4K	TCGA-NH-A5IV-01	Colon Adenocarcinoma	G4S
HIST1H4K	TCGA-MP-A4SW-01	Lung Adenocarcinoma	G4S
HIST1H4K	TCGA-AJ-A3OJ-01	Uterine Endometrioid Carcinoma	G4V

Table S3. List of Oncomine microarray studies with significant deregulation of one or more NAT genes.

Cancer	Study	Up	Down
Bladder	Sanchez-Carbayo et al., J Clin Oncol 2006/02/10	NAA40; NAA50; NAA38; HYPK	None
Brain	Sun et al., Cancer Cell 2006/04/01	NAA40; NAA15; NAA38; NAA16	NAA30; NAA80
Brain	TCGA	NAA40; NAA38; NAA16	None
Breast	Curtis et al., Nature 2012/04/18	NAA10	HYPK
Breast	TCGA	NAA40; NAA60; NAA11	None
Cervix	Scotto et al., Genes Chromosomes Cancer 2008/09/01	NAA10; NAA40; NAA60	NAA80
Cervix	Zhai Cancer Res 2007/11/01	NAA50	None
Colorectal	Hong et al., Clin Exp Metastasis 2010/02/01	NAA10; NAA20; NAA25; NAA80; NAA15; NAA38; HYPK; NAA16	None
Colorectal	Gaedcke et al., Genes Chromosomes Cancer 2010/11/01	NAA10; NAA20; NAA80; NAA11; NAA16	None
Colorectal	TCGA	NAA10; NAA20; NAA50; NAA80; NAA15; NAA25; NAA38; NAA11; NAA16	NAA60
Colorectal	Skrzypczak et al., PLoS One 2010/10/01	NAA10; NAA20; NAA15; NAA25; NAA38	NAA30
Colorectal	Graudens et al., Genome Biol 2006/03/15	NAA10	None
Colorectal	Ki et al., Int J Cancer 2007/11/01	NAA10	None
Colorectal	Sabates-Bellver et al., Mol Cancer Res 2007/12/01	NAA20; HYPK; NAA15	None
Esophagus	Hao et al., Gastroenterology 2006/09/01	HYPK	None
Esophagus	Kim et al., PLoS One 2010/11/30	None	NAA20; NAA16
Gastric	DErrico et al., Eur J Cancer 2009/02/01	NAA10; NAA20; NAA40; NAA50; NAA15; NAA25; NAA38; HYPK; NAA11	None
Gastric	Cho et al., Clin Cancer Res 2011/04/01	NAA50; NAA25	None
Gastric	Chen et al., Mol Biol Cell 2003/08/01	NAA50; NAA25	None
Head&Neck	Estilo et al., BMC Cancer 2009/01/12	NAA20	None
Head&Neck	Sengupta et al., Cancer Res 2006/08/01	NAA50; NAA15	None
Leukemia	Haferlach et al., J Clin Oncol 2010/05/20	NAA10; NAA40; NAA80; NAA25; NAA38	NAA16
Liver	Wurmbach et al.,Hepatology 2007/04/01	NAA20; NAA40	None

Cancer	Study	Up	Down
Liver	Roessler et al., Cancer Res 2010/12/15-Cohort 1	NAA10; NAA40; NAA38	None
Liver	Roessler et al., Cancer Res 2010/12/15-Cohort 2	NAA10; NAA40;NAA38	None
Liver	Mas et al., Mol Med 2008/12/21	None	NAA60; NAA15; HYPK
Lung	Beer et al., Nat Med 2002/08/01	NAA10	None
Lung	Hou et al., PLoS One 2010/04/22	NAA20; NAA40; NAA50; NAA25; NAA38; NAA11	None
Lung	Selamat et al., Genome Res 2012/07/01	HYPK	None
Lung	Bhattacharjee et al., Proc Natl Acad Sci U S A 2001/11/20	None	NAA20
Lymphoma	Compagno et al., Nature 2009/06/04	NAA10; NAA20; NAA60; NAA15; NAA35; NAA38; NAA16	NAA40
Lymphoma	Piccaluga et al., J Clin Invest 2007/03/01	NAA60; NAA15; NAA25; NAA38; NAA38	NAA80
Lymphoma	Brune et al., J Exp Med 2008/09/29	NAA60	None
Myeloma	Zhan et al., Blood 2007/02/15	NAA10; NAA20; NAA50; NAA15; NAA25; NAA35; NAA38; HYPK; NAA16	None
Ovarian	Bonome et al., Cancer Res 2008/07/01	NAA10; NAA38	NAA50; NAA16
Ovarian	Yoshihara et al., Cancer Sci 2009/08/01	NAA40	NAA16
Pancreas	Badea et al., Hepatogastroenterology 2008/11/01	None	NAA16
Pancreas	Pei et al., Cancer Cell 2009/09/08	None	NAA16
Prostate	Tomlins et al., Nat Genet 2007/01/01	NAA30; NAA35	None
Prostate	Lapointe et al., Proc Natl Acad Sci U S A 2004/01/20	HYPK	None
Prostate	Wallace et al., Cancer Res 2008/02/01	None	NAA11; NAA16
Testicular	Sperger et al., Proc Natl Acad Sci U S A 2003/11/11	None	NAA20
Tongue	Talbot Cancer Res 2005/04/15	NAA20	None
Renal	Beroukhim et al., Cancer Res 2009/06/01	NAA38	None
Renal	Jones et al., Clin Cancer Res 2005/08/15	HYPK; NAA16	None

* Significance threshold was Fold change >1.5 between groups and p<0.05

Table S4. DepMap Project Co-dependency Correlations.

	NAA10	NAA20	NAA30	NAA40	NAA50	NAA60	NAA80	NAA15	NAA25	NAA35	NAA38	HYPK	NAA11	NAA16
NAA10	1	0.02	0.00	0.04	-0.02	-0.01	-0.02	0.01	0.01	-0.02	0.03	0.05	0.00	0.00
NAA20		1.00	0.07	0.09	-0.01	0.04	0.00	0.08	**0.20**	0.06	0.04	0.04	0.02	0.01
NAA30			1.00	0.04	0.00	-0.01	-0.07	-0.04	0.10	**0.17**	**0.13**	0.05	-0.05	-0.07
NAA40				1.00	-0.03	0.00	0.08	0.06	0.01	0.07	0.04	0.08	-0.02	0.04
NAA50					1.00	0.02	0.04	-0.10	0.04	0.00	0.03	-0.09	-0.01	0.01
NAA60						1.00	0.03	-0.05	-0.06	-0.03	-0.04	0.01	-0.07	-0.03
NAA80							1.00	0.02	-0.05	-0.03	0.07	0.02	-0.08	-0.04
NAA15								1.00	0.04	0.02	0.05	**0.14**	-0.02	0.04
NAA25									1.00	0.07	-0.02	0.07	-0.02	0.01
NAA35										1.00	0.09	0.10	-0.02	0.00
NAA38											1.00	-0.05	-0.04	0.02
HYPK												1.00	0.08	0.03
NAA11													1.00	0.07
NAA16														1.00

Table shows the co-dependency correlation values calculated for each NAT-NAT pair across the DepMap CRISPR-CAS KO screen. In bold are shown the significant correlations, $p < 0.05$ after Bonferonni correction for multiple testing.

Table S5. Top correlated genes with NAA40 in 107 liver cancer datasets.

Rank	Category	Genes
1	Cell Cycle	ABL1; ANAPC4; ANAPC5; ANAPC7; ATM; ATR; BUB1; BUB1B; BUB3; CCNA2; CCNB1; CCNB2; CCNE1 ;CDC20;CDC25B; CDC45; CDC6; CDC7; CDK4; CHEK1 HDAC1; MCM2;MCM3; MCM4;MCM5;MCM6; MCM7;ORC6;PRKDC; SMC3; TP53; TTK;YWHAB
2	DNA Replication	FEN1; LIG1; MCM2;MCM3;MCM4;MCM5;MCM6;MCM7;POLA1; POLA2;POLD1;POLD3;PRIM2;RFC4
3	RNA transport	AAAS; ACIN1; ALYREF; CASC3; CLNS1A; DDX39B; EIF3B; EIF4G3; ELAC2; KPNB1; NUP133; NUP188; NUP205; NUP210; NUP43; NUP62; NUP85; NUP93; POP1; RNPS1; TACC3; THOC2; THOC5; UBE2I; UPF3B; XPO5
4	Spliceosome	ACIN1; ALYREF; DDX23; DDX39B; DDX42; EFTUD2; HNRNPA3; HNRNPM; LSM2; PRPF19; PRPF31; PRPF4; PRPF6; RBM25; SART1; SF3B2; SNRNP200; SNRNP40; SNRNP70; SNRPA; SRSF1; THOC2; U2AF2
5	mRNA surveillance	ACIN1; ALYREF; CASC3; CPSF1; CPSF6; CPSF7; DAZAP1; DDX39B; MSI2; PABPN1; PPP1CA; PPP2R1A; RNPS1; SMG5; SYMPK; UPF3B; WDR82
6	Pyrimidine Metabolism	CAD; DTYMK; ENTPD4; ENTPD6; ITPA; NME6; POLA1; POLA2; POLD1; POLD3; POLR1A; POLR2B; POLR2G; POLR3A; PRIM2; RRM1; TYMS

Genes found within top six enriched KEGG categories for genes correlating with NAA40 across 107 liver cancer datasets (see **Fig.5E**)
